# Influence of Anthropometric Characteristics and Muscle Performance on Punch Impact

**DOI:** 10.3390/sports13080281

**Published:** 2025-08-21

**Authors:** Manuel Pinto, João Crisóstomo, Christopher Kirk, Javier Abián-Vicén, Luís Monteiro

**Affiliations:** 1 CIDEFES, Faculdade de Educação Física e Desporto, Lusófona University, 1749-024 Lisbon, Portugal; jcpcrisostomo@gmail.com (J.C.); luis.monteiro@ulusofona.pt (L.M.); 2 Sport and Physical Activity Research Centre, Sheffield Hallam University, Collegiate Crescent, Sheffield S10 2BP, UK; c.kirk@shu.ac.uk; 3 Performance and Sport Rehabilitation Laboratory, Faculty of Sport Sciences, University of Castilla-La Mancha, Avda. Carlos III s/n, 45071 Toledo, Spain; 4 ICPOL Research Center, Higher Institute of Police Sciences and Internal Security, 1300-352 Lisbon, Portugal

**Keywords:** combat sport, functional strength development, exercise prescription for power training, anthropometry

## Abstract

Despite the known relevance of punch impact in boxing, limited evidence exists regarding how anthropometric and muscle performance variables contribute to it. This study investigated the relationship between anthropometric characteristics, muscle power and strength performance, and punch impact power in 69 boxing practitioners (mean ± SD age: 27.0 ± 6.1 years). Anthropometric variables (body height (BH), armspan (AS), body mass (BM)) and muscle power and strength tests (countermovement jump (CMJ), one repetition maximum in bench press (1RM BP), and handgrip strength (HS)) were assessed. Punch impact power was assessed with PowerKube (PK), a specific device designed to measure punch impact power. Punch impact power was positively correlated with BH, AS, and BM. Linear regression indicated that BH and AS explained about 36% of the variance in Straight punch impact power and 30–34% in Hook punch impact power. BM showed weaker predictive capacity, explaining 10% of the variance in Straight punch impact power and 11% in Hook punch impact power. When comparing punch impact power differences across groups with varying BH, AS, and BM, it was found that groups with High BH exhibited higher punch impact power than the groups with Low and Medium BH for both Straight and Hook punches. For AS, the High AS group also demonstrated higher punch impact power, with similar trends for BM, where significant differences were observed only between the High and Low BM groups. Additionally, our findings confirm significant relationships between anthropometric characteristics, muscle power, and strength performance. These findings highlight the importance of a comprehensive assessment of anthropometric profiles, alongside muscle power and strength evaluations, to better predict punch impact power. This approach provides valuable insights for boxing training and may also inform exercise programming for the general population.

## 1. Introduction

Boxing is a striking combat sport with a long tradition and global relevance, where understanding the characteristics that influence practitioners’ punching performance is of great importance. The sport of boxing is characterized by the application of striking techniques using the upper limbs (punches), aiming either to achieve temporary incapacitation of the opponent or to accumulate points [1,2]. The primary objective of boxing is to land an effective strike on the opponent without receiving a counterstrike, essentially controlling the competition [3].

Boxing strikes are categorized into three main types: (a) Straight punches (delivered in a frontal direction), (b) Hooks (executed with lateral movements), and (c) Uppercuts (performed with vertical motions) [4]. Among these, Straight punches and Hooks are the most commonly employed techniques in boxing competitions [5,6,7]. To increase the likelihood of concluding a match by incapacitating the opponent, the impact of punches has been extensively analyzed [8,9,10]. The impact power of punches has been measured using scientifically validated instruments, such as PowerKube (PK), which is specifically designed to evaluate punch impact power. This portable 3.0 kg device integrates two high-precision accelerometers that relay information to proprietary software for precise measurements [11].

The effectiveness of punch impact is a complex movement involving both upper and lower body musculature, requiring coordinated action between agonist and antagonist muscles [12,13,14]. Numerous studies have explored the relationship between muscle performance (strength and power output) and punch impact. Significant correlations have been reported between performance in strength and power tests—such as the one-repetition maximum bench press (1RM BP), countermovement jump (CMJ), and handgrip strength (HS)—and the impact of boxing punches [15,16,17].

Anthropometric characteristics, especially body height (BH), armspan (AS), and body mass (BM), have been widely recognized as critical factors influencing athletes’ combat strategies [18,19]. Despite their importance in shaping combat strategies, the relationship between anthropometric characteristics and success in boxing remains unclear, with existing evidence being inconclusive [20,21]. While boxing athletes with higher BM generally produce punches with greater impact, this variable is not solely determined by BM [22,23,24]. Comparative studies across BM categories have highlighted nuances in this relationship, showing that lighter athletes often generate greater impact in strikes with longer trajectories due to higher strike velocities and extended acceleration times. In contrast, heavier athletes tend to excel in producing high impact during shorter strikes [24].

Although the literature on the influence of anthropometric characteristics on punch impact power is extensive, it remains unclear how or whether BH, AS, and BM influence the impact of different punch types. Despite considerable research on punch impact over the past two decades, to the best of our knowledge, no study has systematically examined the relationship between anthropometric characteristics and punch impact power levels. While this study focuses on elite boxers, understanding the relationship between anthropometrics and power generation has broader implications for general populations, particularly in self-defense training and fall prevention programs for older adults.

## 2. Methods

### 2.1. Participants

An a priori power analysis (G*Power 3.1.9.4; Kiel University, Kiel, Germany) determined that a total sample size of 64 participants was required based on the following parameters: correlation point biserial model, statistical power of 0.80, α-value of 0.05, and an effect size of 0.30. Consequently, 69 male boxing practitioners (age: 27.0 ± 6.1 years; years of practice: 3.6 ± 4.3 years) from the same boxing gym volunteered to participate in the study.

Participants had been training in boxing for at least one year, at a frequency of at least twice per week, with each session lasting approximately 60 min, in the 4 weeks preceding the testing period. None of the participants reported muscle or joint injuries in the 3 months prior to recruitment, nor were they taking any drugs, medications, or dietary supplements that could interfere with the experimental procedures.

This study was approved by the Local Ethics Committee of Lusófona University (approval number AB3025), and all procedures were conducted in accordance with the Declaration of Helsinki for human experimentation [25]. Participants received detailed information about the study design, associated risks, and their right to withdraw at any stage of the process. Written informed consent was obtained from all participants before the commencement of the study. Recruitment was carried out through newsletters, publications on the gym website, and social media platforms (Facebook and Instagram).

Potential moderators included performance metrics (CMJ, 1RM BP, and HS). These variables were analyzed to assess their influence on the relationships between anthropometric characteristics, muscle performance, and punch impact power.

### 2.2. Procedure

**Figure 1:**
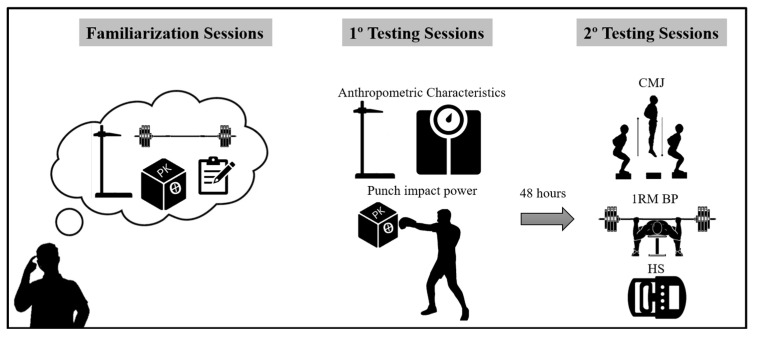
Schematic representation of the study design.

Both testing sessions were conducted by the same researcher and two boxing coaches at the boxing gym, over two days between 8:00 and 11:30 a.m. Participants were instructed to abstain from consuming caffeine, alcohol, or engaging in strenuous physical activity during the 24 h preceding each test session. In the week prior to experimentation, athletes conducted two familiarization sessions with the testing procedures to minimize the learning effect. On the first day, anthropometric measurements were obtained (8:00–10:00 a.m.), followed immediately by an assessment of punch impact power using the PK device (Strike Research Ltd., Norwich, England) (10:00–11:30 a.m.). The second session, conducted after a minimum of 48 h to mitigate potential fatigue effects, focused on physical performance assessment (CMJ, 1RM BP, and HS). Participants were required to avoid any training or strenuous activities during the 48 h preceding each testing session to ensure reliable performance outcomes. The experimental procedures are detailed in Figure 1.

### 2.3. Measures

#### 2.3.1. Anthropometric Characteristics Analysis

BH was measured to the nearest 0.1 cm using a stadiometer (Seca Model 217; Birmingham, UK), with participants barefoot. BM was assessed using an electronic scale (Seca 713; Hamburg, Germany) with a capacity of 150 kg and accuracy to the nearest 100 g. Participants wore light athletic clothing (shorts and a t-shirt) and stood barefoot, without movement, on the device. AS was measured to the nearest 0.1 cm using a metal tape measure. Participants stood upright with arms fully extended horizontally at shoulder height, palms facing forward. The body mass index (BMI) was calculated using the formula: BMI = BM/BH^2^ (kg·m^−2^).

Anthropometric measurements were collected by a single trained observer to minimize measurement errors [26,27]. The observer’s reliability was confirmed through a test-retest process, achieving high consistency with a reliability coefficient of 1 for BH and BM measurements and a reliability coefficient of 0.98 for AS measurements, across two series of intra-observer measurements [27,28]. Reliability, as defined by Malina et al. [29], is calculated using the following formula: r = S1^2^/(S1^2^ + S2^2^), where ‘r’ represents the reliability coefficient (ranging from 0 to 1), S1^2^ is the variance from the first test session, and S2^2^ is the variance from the second test session.

#### 2.3.2. Measurement of Punch Impact Power

Punch impact power was measured using the PK. Before the assessment, all participants received instructions and performed a familiarization session with the PK. The procedures included a general warm-up; the use of standardized gloves (10 oz) and bandages (2.5 m); technical instructions on strike execution; a specific warm-up with shadow boxing; individual adjustments of the PK to ensure the target was at chin height for precise measurement of both Straight punches and Hooks; and progressive effort tests to accustom participants to the instrument [30].

The general warm-up consisted of the following activities: 90 s of jumping rope, 10 forward and backward shoulder and elbow circles (each side), 10 front and side leg swings (each leg), 10 lunges per leg, and 45 s of boxing footwork (front, back, left, right) on command.

Participants received 3 min of supervised technical practice under a coach’s guidance. This included imitating the coach’s movements and receiving corrections to ensure proper technique. Coaches also identified and classified the type of punches delivered by each practitioner. Then, participants completed 3 rounds of shadow boxing, each lasting approximately 3 min, with 5 min of rest between rounds. Each round involved ~180 strikes (3 strikes every 3 s), executed in response to auditory signals from the coach.

The PK was individually adjusted for each participant and strike type: Straight punches: participants assumed a standard boxing guard position, standing at a self-selected arm’s length from the PK, with the target at chin height. Hook punches: participants stood next to the PK, with the fist in contact with the target at a ~90-degree elbow angle.

For the maximum effort strikes, participants executed 6 Straight punches and 6 Hook punches with each hand, totaling 24 strikes. To prepare, they performed 4 strikes at 50% perceived maximum effort, followed by the maximum effort strikes. The order of strikes alternated between hands and punch types, with a minimum rest period of 3 s between punches to allow participants to regain proper positioning. Relative punch impact power was calculated using the formula: Relative punch impact power = absolute punch impact power (W)/body mass (kg).

#### 2.3.3. Power and Strength Tests

Participants performed the CMJ on an electronic contact platform (Chronojump Contact Platform A2, Bosco Systems, Barcelona, Spain) to measure the maximum vertical jump height. Athletes were instructed to place their hands on their hips and stand with their feet shoulder-width apart. They performed a countermovement by bending their knees until reaching a 90-degree angle before initiating the concentric phase of the CMJ. Three trials were completed, with 10–15 s of rest between them, and the best performance trial was used for the subsequent statistical analysis [31].

CMJ jump height, along with the athlete’s body mass, was used to calculate absolute peak power (W) and relative peak power (W·kg^−0.67^). Absolute peak power was determined using the equation by Sayers et al. [32]:

Absolute peak power (W) = 60.7 × jump height (cm) + 45.3 × body mass (kg) − 2055.

Relative peak power, derived using allometric scaling, was calculated using the formula: Relative peak power (W·kg^−0.67^) = absolute peak power (W)/body mass (kg)^0.67^.

For the 1RM BP test, a normal standard Olympic bar and plates were used for the lifts. The bench press procedure was standard ‘‘touch-and-go’’ protocol [33]. Participants warmed up for the bench press test with 5 min of light cycling on a leg ergometer at a self-selected intensity. The specific warm-up began with 5 repetitions at approximately 50% of an estimated 1RM BP, based on prior experience or evaluator guidance. After a 1 min rest, participants performed 3 repetitions with 60–70% of the estimated load. Following a 2 min rest, 2 repetitions were performed at 80–85% of the estimated load. Another 2 min rest preceded the first 1RM BP attempt. The first attempt was performed with a load near 90–95% of the estimated 1RM BP. If successful, the load was increased by 2–5% (minimum increase in weight was 3 kg); if unsuccessful, it was reduced by 2–5%. Participants were allowed 3–6 attempts to determine their 1RM BP, depending on fatigue and success rates. Rest intervals of 3–5 min were provided between attempts to ensure recovery and minimize fatigue. A successful lift required the barbell to touch the chest, pause slightly, and be raised to full arm extension using correct technique [34,35].

Relative 1RM BP was calculated using the formula: Relative 1RM BP = 1RM BP (kg)/body mass (kg).

Handgrip strength was assessed using a dynamometer (Takei Physical Fitness Test, TKK 5001, GRIP–A, Tokyo, Japan) to measure the strength of hand and forearm muscles. Participants were seated upright with hips and knees flexed at 90°, feet flat on the floor, and the tested arm positioned at the side without touching the torso. The elbow was flexed at 90°, forearm in a neutral position, and wrist positioned between 0° and 30° of extension, with 0° to 15° of ulnar deviation. Participants were instructed to squeeze the dynamometer with maximum force for 3–5 s, avoiding movement of the arm or trunk. The test was performed three times for each hand, with 60 s intervals between attempts to prevent fatigue. The highest recorded value from each hand was used for analysis [36,37].

### 2.4. Statistical Analyses

Data was analyzed using IBM SPSS software (version 25.0; IBM Corp, Armonk, NY, USA). The Shapiro–Wilk test revealed the normal distribution of all the considered variables. Therefore, data are presented as mean ± standard deviation [95% confidence interval]. Pearson’s correlation coefficient (r) was used to examine relationships between anthropometric characteristics, muscle power and strength performance, and punch impact power. The magnitude of correlations was assessed using the following benchmarks: <0.1: trivial; 0.1–0.3: low; 0.3–0.5: moderate; 0.5–0.7: large; 0.7–0.9: very large; >0.9: nearly perfect; =1: perfect [38]. Based on the correlation coefficients, simple linear regression was used to model the relationship between a single dependent variable (punch impact power) with one independent variable (anthropometric characteristics or muscle power and strength performance). Regarding group comparisons, one-way ANOVA tests were used to compare mean punch impact power between groups, and partial eta squared (ηp^2^) was calculated as a measure of effect size. The magnitude of ηp^2^ was interpreted as follows: 0.01: small; 0.06: medium; 0.14: large [39]. Terciles were created for the anthropometric variables, and mean punch impact power was compared across these terciles using a one-way ANOVA test with Bonferroni post-hoc tests. Participants were divided into terciles (low, medium, high) for each characteristic: BH, AS, and BM. Statistical significance was accepted when *p* < 0.05.

## 3. Results

Anthropometric characteristics, muscle power and strength performance, and punch impact power performance are provided in Table 1.

Correlations between anthropometric characteristics, muscle power and strength performance, and punch impact power performance are reported in Table 2, Table 3 and Table 4.

Simple linear regression models to predict the main specific punch impact power based on anthropometric characteristics and muscle power and strength performance are reported in Table 5 and Table 6.

**Figure 2:**
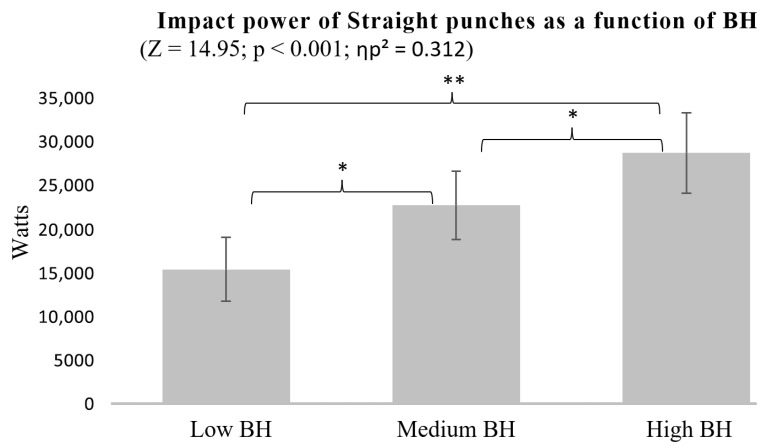
Impact power of Straight punches as a function of BH. * *p* < 0.05, ** *p* < 0.01.

The analysis of Straight punch impact power across groups with different BH revealed statistically significant differences. The High BH group consistently demonstrated greater impact power, outperforming both the Low (*p* < 0.001) and Medium BH groups (*p* = 0.043). Additionally, the Medium BH group exhibited significantly higher impact power than the Low BH group (*p* = 0.013), indicating a progressive increase in impact power with greater body height (Figure 2).

**Figure 3:**
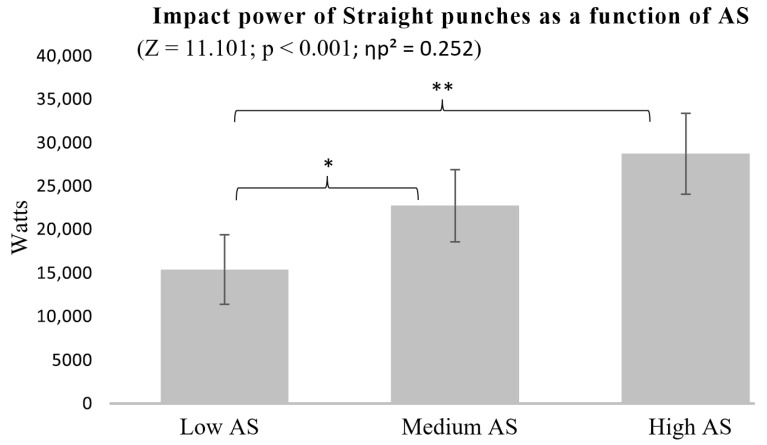
Impact power of Straight punches as a function of AS. * *p* < 0.05, ** *p* < 0.01.

When analyzing Straight punch impact power across different AS groups, the results followed a similar pattern. The High AS group demonstrated significantly greater power than the Low AS group (*p* < 0.001), while the Medium AS group also outperformed the Low AS group (*p* = 0.012). However, no significant differences were observed between the High and Medium AS groups, which may indicate that a longer AS is beneficial up to a point, after which other factors might influence impact power (Figure 3).

**Figure 4:**
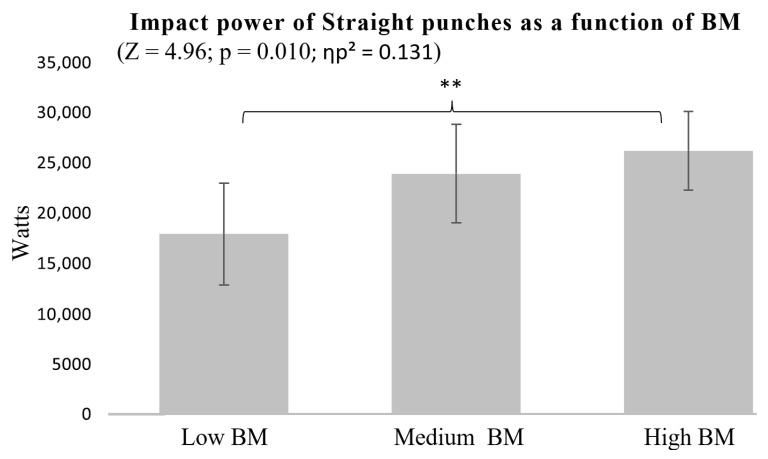
Impact power of Straight punches as a function of BM. ** *p* < 0.01.

The analysis of BM and Straight punches revealed a significant difference between the High and Low BM groups (*p* = 0.010), while the Medium BM group did not differ significantly from the other two (Figure 4).

**Figure 5:**
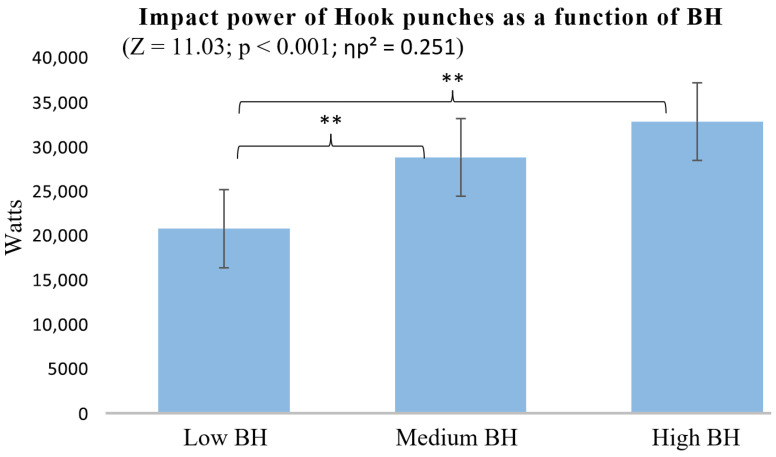
Impact power of Hook punches as a function of BH. ** *p* < 0.01.

Regarding the Hook punch impact power across groups with different BH, the High BH group produced significantly greater impact power than the Low BH group (*p* < 0.001). Although the Medium BH group also exhibited greater power than the Low BH group (*p* = 0.010), no significant difference was found between the High and Medium BH groups, suggesting that body height may have a diminishing effect on impact power beyond a certain threshold (Figure 5).

**Figure 6:**
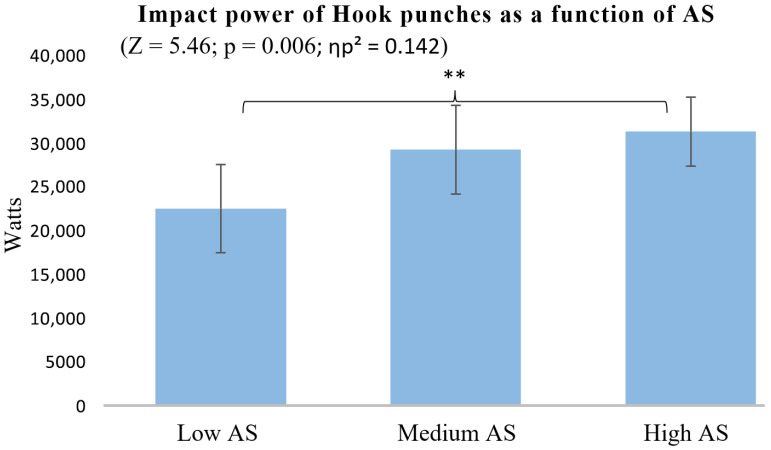
Impact power of Hook punches as a function of AS. ** *p* < 0.01.

For the analysis of AS and Hook punch impact power, the High AS group again exhibited significantly greater power than the Low AS group (*p* = 0.007). However, in contrast to the Straight punch results, no significant differences were found between the other groups, suggesting that AS may play a less decisive role in Hook punch execution (Figure 6).

**Figure 7:**
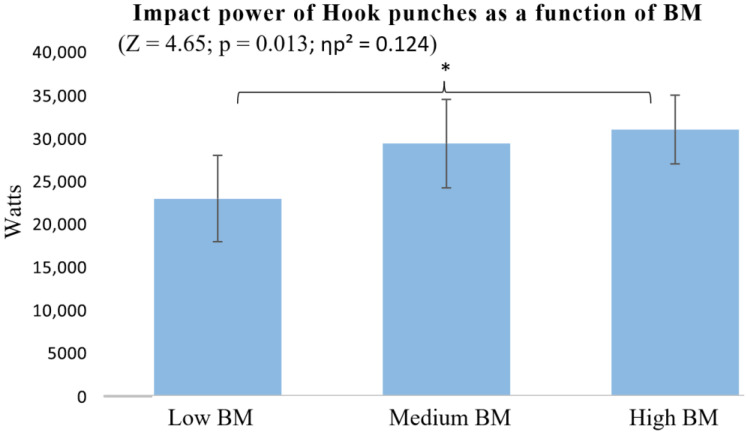
Impact power of Hook punches as a function of BM. * *p* < 0.05.

Finally, the analysis of BM and Hook punch showed significant differences only between the High and Low BM groups (*p* = 0.015) (Figure 7).

## 4. Discussion

This study explored the relationships between anthropometric characteristics (BH, AS, and BM), muscle power, and strength performance (CMJ, 1RM BP, and HS) with punch impact power in male boxing practitioners. Punch impact power was assessed using the PK, a specific instrument widely used in similar studies [17]. Previous research reports mean punch impact power ranging from 6781.6 ± 2178.9 W to 22014 ± 1336 W [40,41,42]. However, these studies predominantly evaluated Straight punch impact power. Expanding upon this, our study assessed the two most common boxing techniques: the Straight punch and the Hook punch [5,6,7].

In our sample, punch impact power for the Straight punch showed means of 22,677.7 ± 9771.2 W, while the Hook punch showed means of 27,822.8 ± 9971.3 W (Table 1). These results indicated higher levels of impact in Hook punches, aligning with previous studies [7,15,43]. Interestingly, while relationships between muscle power or strength performance and punch impact power have been investigated [15,16,17], no prior studies have comprehensively examined the interplay between anthropometric characteristics, muscle power, and strength performance with punch impact power. To our knowledge, this makes our study the first to address these associations in male boxing practitioners (Table 2, Table 3, Table 4, Table 5 and Table 6).

In line with other combat sports studies, our findings supported the first hypothesis, confirming significant relationships between anthropometric characteristics, muscle power, and strength performance [44,45]. Specifically, all muscle power and strength performance tests (CMJ, 1RM BP, and HS) demonstrated positive correlations with BH and AS (Table 2). These findings underscore the critical role of BH and AS, alongside physical performance measures, in determining punch impact power.

Curiously, BM showed fewer positive significant correlations with muscle power and strength performance variables compared to BH and AS (Table 2). This finding contrasts with the equation by Sayers et al. [32], which is used to calculate the absolute peak power of jumping performance and is based on the achieved vertical height and BM. Therefore, the relationship between these two parameters and their association with other anthropometric characteristics may influence the correlation analyses. Consequently, CMJPpeak showed a very large correlation magnitude with BM (and significant). In contrast, the muscle strength performance, expressed in terms of Relative 1RM BP, showed a moderate, significant negative correlation with BM (r = −0.420). The observation that heavier male boxing practitioners were more powerful in the lower limbs but demonstrated less relative strength in the upper limbs could be attributed to several factors. Heavier individuals generally have more muscle mass, which contributes to greater absolute power in movements such as jumps. However, the additional body mass can also lower relative strength (strength-to-body mass ratio) in upper-body exercises like the 1RM bench press, since the extra mass does not contribute to the strength needed for upper-body lifts. This trade-off between power and relative strength is commonly observed in athletes with higher body mass, where lower-body power may benefit from extra weight, while upper-body strength becomes less efficient.

Regarding BMI, this variable showed a significant positive correlation with CMJPpeak (r = 0.444) and significant negative correlations with CMJHeight (r = −0.444), Relative CMJPpeak (r = −0.370), and Relative 1RM BP (r = −0.561) (Table 2). These results suggest that while a higher BMI may contribute positively to absolute power output in the CMJ, it negatively impacts performance in metrics relative to BM, such as jump height, relative peak power, and relative strength. This highlights the importance of considering both absolute and relative measures when evaluating physical performance.

The second hypothesis was validated, as correlations between punch impact power and anthropometric characteristics were identified. Specifically, both the Straight punch and Hook punch exhibited significant positive correlations with BH (r = 0.597 and r = 0.583, respectively) and AS (r = 0.601 and r = 0.550, respectively). Significant positive correlations were also observed with BM (r = 0.326 for Straight punch and r = 0.362 for Hook punch) (Table 3). However, it is worth noting that these correlations may be influenced by confounding factors, such as the potential relationship between BM and BH, given that taller participants are often heavier in this study.

Regarding BM, these findings align with the study by Mosler et al. [46], which demonstrated a significant positive correlation (ϱ = 0.520) between BM and effective mass. This indicates that greater BM enhances the effective utilization of BM to generate punch force. While few studies have directly explored the relationships between BH or AS and punch impact power, research in striking combat sports has emphasized the importance of these variables [24,47]. Podhurskyi [24] highlighted that in Muay Thai, a striking combat sport, differences in the power of technical movements are influenced not only by BM but also by limb dimensions and the leverage exerted during striking actions. Additionally, the significance of BH has been examined not only as a determinant of athletic performance but also as a potential factor in competition category divisions. Dubnov-Raz et al. [47] proposed incorporating BH alongside BM in classification systems to mitigate the prevalence of eating disorders among young athletes while providing competitive advantages in striking combat sports.

In our study, BMI showed significant negative correlations with Straight punch impact power (r = −0.314) and Hook punch impact power (r = −0.316) (Table 3). While BM alone may enhance punch impact power, BMI (which accounts for BH) might highlight limitations in relative performance efficiency.

These findings suggest that BH, AS, and BM play crucial roles in generating punch impact power, with larger anthropometric dimensions generally contributing to improved striking performance. However, the negative correlations with BMI emphasize the need to consider both absolute and relative performance metrics in combat sports. It is important to note that simple linear regression models revealed that BH and AS explained only ~36% of the variance in Straight punch impact power and 30–34% of the variance in Hook punch impact power (Table 5). BM showed weaker predictive capacity, accounting for only 10% of the variance in Straight punch impact power and 11% in Hook punch impact power.

The third hypothesis was confirmed, as correlations between punch impact power, muscle power, and strength performance were observed. Specifically, both the Straight punch and Hook punch demonstrated moderate to large correlations across all muscle power and strength performance variables, including CMJ, 1RM BP, and HS (Table 4).

Previously, Loturco et al. [12] reported that CMJ height had a significant positive correlation with punch impact force in amateur boxers, with correlation coefficients ranging from 0.67 to 0.85 across different punch types. In our study, linear regression models indicated that CMJheight, CMJPpeak, and Relative CMJPpeak accounted for 27%, 31%, and 30% of the variance in Straight punch impact power, respectively, and 20%, 31%, and 32% of the variance in Hook punch impact power (Table 5 and Table 6). This relationship can be explained by the ability of the lower limbs to generate greater force and rate of force development (RFD) into the ground, which enhances kinetic chain transfer through the body, ultimately resulting in greater punch impact power. These findings support the established role of lower-body power in generating punch impact [14,48].

Additionally, López-Laval et al. [16] demonstrated that the velocity achieved at 80% of 1RM BP showed a significant positive correlation (r = 0.815) and could explain up to 75% of the variance in rear arm punch velocity among professional boxers. While our study focused exclusively on maximal upper-body strength using 1RM BP, the results were consistent with López-Laval et al. [16] findings. Moderate to large correlations were observed between 1RM BP and punch variables, including Straight punch impact power, Relative Straight punch impact power, Hook punch impact power, and Relative Hook punch impact power (Table 4). Moreover, our linear regression models revealed that 1RM BP could explain 38% of the variance in Straight punch impact power and 51% in Hook punch impact power. Similarly, but to a lesser extent, Relative 1RM BP explained 15% of the variance in Straight punch impact power and 19% in Hook punch impact power (Table 5 and Table 6).

Our findings also align with those of Bružas et al. [49] and Čepulėnas et al. [50], who demonstrated significant positive correlations between HS and punching impact. Bružas et al. [49] reported significant positive correlations for the Straight punch (r = 0.740) and for the Hook punch (r = 0.630). Similarly, our study found significant positive correlations between HS and Straight punch impact power (r = 0.518) as well as Hook punch impact power (r = 0.648) (Table 4). Furthermore, linear regression models showed that HS explained 27% of the variance in Straight punch impact power and 42% in Hook Punch impact power (Table 5 and Table 6).

The findings support the fourth hypothesis, indicating that BH influences punch impact power (Figure 2 and Figure 5). Participants with High BH tended to generate higher impact forces in both Straight and Hook punches. Differences were also observed among those with Medium and Low BH, suggesting a progressive effect. The ANOVA results indicated large effect sizes for the influence of BH on punch impact power in both Hook (ηp^2^ = 0.251) and Straight punches (ηp^2^ = 0.312), values well above the threshold for a large effect, indicating that a substantial proportion of the variance in impact power can be explained by differences in BH.

A similar pattern emerged when considering AS, with longer reach being associated with greater punch impact power (Figure 3 and Figure 6). However, the difference between individuals with High and Medium AS was less pronounced, particularly for Hook punches. Still, effect sizes for AS were large in both Hook (ηp^2^ = 0.142) and Straight punches (ηp^2^ = 0.252), reinforcing the importance of reach in generating impact power.

In contrast, BM appeared to play a more limited role, with significant differences mainly observed between individuals with High and Low BM (Figure 4 and Figure 7), while those with Medium BM showed no clear advantage. The effect sizes for BM (ηp^2^ = 0.124–0.131) fell within the medium range, indicating a more modest contribution to punch impact compared to BH and AS. These results highlight the strong influence of BH and AS on punching performance, while the effect of BM appears comparatively moderate and possibly more situational.

These results suggest that BM alone has a limited impact on punch impact power, emphasizing that punch performance involves a complex interplay of factors. Effective punching relies not only on anthropometric characteristics but also on the coordinated contribution of upper- and lower-body muscles, proper agonist–antagonist cooperation, and technical skill level [1,17,18].

While BH, AS, and BM influence punch impact power to some extent, they do not fully capture the ability to generate force. These findings highlight the importance of a comprehensive assessment of anthropometric profiles alongside evaluations of muscle power and strength performance, experience, and skill level to better understand and enhance sport-specific characteristics in practitioners.

## 5. Limitations

This study provides valuable insights into the relationships between anthropometric characteristics, muscle power, and punch impact power in male boxing practitioners; however, several limitations must be acknowledged: First, the study sample consisted of boxing practitioners with limited experience, and with different years of practice, which may not represent the broader population of boxers, including elite or professional athletes. This restricts the applicability of the findings to more advanced or novice practitioners. Future research should include participants with a wider range of experience levels to enhance the external validity of the results. Second, all participants were recruited from the same gym and followed a standardized training program. While this consistency reduces variability, it limits the generalizability of the findings to practitioners from other training environments, styles, or methodologies. Including participants from diverse gyms and training regimens could provide a more comprehensive understanding of the factors influencing punch impact power. Third, the cross-sectional design of the study limits the ability to infer causal relationships between the analyzed variables. Longitudinal studies tracking changes in anthropometric characteristics, physical performance, and punch impact power over time would provide deeper insights into the developmental pathways of boxing practitioners. By addressing these limitations, future research could provide a more nuanced understanding of the factors contributing to punch impact power and optimize training strategies for boxing practitioners.

## 6. Conclusions

This study demonstrates that anthropometric characteristics, particularly greater BH, AS, and BM, significantly contribute to punch impact power in male boxing practitioners. Notably, lower and upper-body performance, assessed through CMJ, 1RM BP, and HS, emerged as critical determinants of punch impact power, underscoring the importance of a holistic approach that integrates anthropometric profiling with physical performance assessments. Additionally, the interplay between relative and absolute measures, such as BMI and relative strength, highlights the complexity of punch impact power generation, emphasizing the need for tailored training strategies that address both absolute force production and efficiency relative to BM. The findings further support the development of evidence-based training interventions aimed at maximizing punch impact through targeted enhancements in power and strength performance. These findings may inform exercise prescription not only for combat sports athletes but also for recreational practitioners seeking to improve functional strength and power. Future studies should adopt experimental designs to validate these relationships across broader populations, including athletes with diverse anthropometric profiles, skill levels, and experience. Such research could provide deeper insights into optimizing training methodologies and improving sport-specific outcomes in boxing and related striking combat sports.

## 7. Practical Application

The findings of this study provide valuable insights for coaches and strength and conditioning professionals aiming to enhance punch impact power. Specifically, the study highlights two key practical applications: First, training programs should focus on improving upper-body strength, lower-body power, and handgrip strength, as these variables were strongly correlated with punch impact power. Additionally, exercises designed to enhance coordination and agonist–antagonist cooperation should be incorporated to optimize force transfer during punches. Plyometric training for lower-body explosiveness and resistance training for upper-body strength can significantly enhance the power of both Straight and Hook punches. This integrated approach addresses the relationship between anthropometric characteristics and physical performance factors that impact punching ability. Furthermore, considering the observed negative correlations between BMI and relative performance metrics (e.g., Relative 1RM BP), training programs should aim to balance improvements in absolute power with relative performance, particularly for athletes with higher BMI. Second, in addition to BM, other anthropometric dimensions such as BH and AS should be considered in competition classification criteria. These factors significantly influence punch impact power and offer a more equitable basis for matching opponents. Furthermore, it is essential to address eating disorders and promote the overall well-being of athletes to ensure sustainable and healthy performance gains.

## Figures and Tables

**Table 1:** Descriptive statistics of anthropometric characteristics, muscle power and strength performance, and punch impact power in male boxing practitioners (values are presented as mean ± standard deviation [95% confidence interval], N = 69).

Variable	Mean ± SD [95% CI]
Anthropometric characteristics	
BH (cm)	174.5 ± 8.5 [172.4–176.5]
AS (cm)	175.9 ± 9.5 [173.6–178.2]
BM (kg)	78.97 ± 12.99 [75.86–82.10]
BMI (kg·m^−2^)	26.90 ± 3.64 [25.03–26.78]
Muscle power and strength performance	
CMJheight (cm)	32.88 ± 6.16 [31.40–34.36]
CMJPpeak (W)	3518.6 ± 687.5 [3353.4–3683.7]
CMJPpeak (W·kg^−0.67^)	44.52 ± 5.05 [43.31–45.74]
1RM BP (kg)	74.35 ± 16.78 [70.32–78.38]
Relative 1RM BP (kg/kg)	0.96 ± 0.24 [0.90–1.01]
HS (kg)	40.47 ± 9.64 [38.15–42.78]
Punch impact power	
Straight punch (W)	22,677.7 ± 9771.2 [20,330.4–25,024.9]
Straight punch (W/kg)	287.85 ± 126.57 [257.44–318.25]
Hook punch (W)	27,822.8 ± 9971.3 [25,427.4–30,218.2]
Hook punch (W/kg)	353.44 ± 123.21 [323.84–383.04]

**Notes:** 1RM BP: 1 repetition maximum bench press; AS: armspan; BH: body height; BM: body mass; BMI: body mass index; CMJ: countermovement jump; HS: handgrip strength; Ppeak: peak power.

**Table 2:** Correlation coefficients (r) between anthropometric characteristics and muscle power and strength performance in male boxing practitioners (N = 69).

Anthropometric Characteristics					
		BH (cm)	AS (cm)	BM (kg)	BMI (kg·m^−2^)
Muscle power and strength performance	CMJheight (cm)	0.586 **	0.497 **	−0.032	−0.444 **
	CMJPpeak (W)	0.792 **	0.730 **	0.839 **	0.444 **
	Relative CMJPpeak (W·kg^−0.67^)	0.603 **	0.517 **	0.033	−0.370 **
	1RM BP (kg)	0.488 **	0.432 **	0.211	−0.080
	Relative 1RM BP (kg/kg)	0.105	0.071	−0.420 **	−0.561 **
	HS (kg)	0.526 **	0.435 **	0.408 **	0.117

**Notes:** 1RM BP: 1 repetition maximum bench press; AS: armspan; BH: body height; BM: body mass; BMI: body mass index; CMJ: countermovement jump; HS: handgrip strength; Ppeak: peak power. ** *p* < 0.01.

**Table 3:** Correlation coefficients (r) between anthropometric characteristics and punch impact power in male boxing practitioners (N = 69).

Anthropometric Characteristics					
		BH (cm)	AS (cm)	BM (kg)	BMI (kg·m^−2^)
Muscle power and strength performance	Straight punch (W)	0.597 **	0.601 **	0.326 **	−0.020
	Straight punch (W/kg)	0.414 **	0.422 **	−0.035	−0.314 **
	Hook punch (W)	0.583 **	0.550 **	0.362 **	−0.027
	Hook punch (W/kg)	0.369 **	0.340 **	−0.058	−0.316 **

**Notes:** AS: armspan; BH: body height; BM: body mass; BMI: body mass index. ** *p* < 0.01.

**Table 4:** Correlation coefficients (r) between muscle power and strength performance, and punch impact power in male boxing practitioners (N = 69).

Muscle Power and Strength Performance							
		CMJheight (cm)	CMJPpeak (W)	Relative CMJPpeak (W·kg^−0.67^)	1RM BP (kg)	Relative 1RM BP (kg/kg)	HS (kg)
**Punch impact power**	Straight punch (W)	0.513 **	0.559 **	0.545 **	0.616 **	0.381 **	0.518 **
	Straight punch (W/kg)	0.556 **	0.273 *	0.579 **	0.573 **	0.577 **	0.380 **
	Hook punch (W)	0.445 **	0.552 **	0.463 **	0.714 **	0.436 **	0.648 **
	Hook punch (W/kg)	0.491 **	0.218	0.495 **	0.686 **	0.684 **	0.511 **

**Notes:** 1RM BP: 1 repetition maximum bench press; CMJ: countermovement jump; HS: handgrip strength. * *p* < 0.05, ** *p* < 0.01.

**Table 5:** Simple linear regression models to estimate main Straight punch impact power performance from anthropometric characteristics muscle power and strength performance in male boxing practitioners (N = 69).

	Equations	R2	Adjusted R2	SEE	*p*
Straight punch	−96,758.37 + 68,458.93 BH (cm)	0.356	0.347	7896.9	<0.001
	−85,817.354 + 61,668.257 AS (cm)	0.361	0.351	7896.4	<0.001
	3296.660 + 245.397 BM (kg)	0.107	0.093	9304.7	0.006
	24,061.723 − 53.430 BMI (kg·m^−2^)	0.001	−0.015	9841.8	0.851
	−4080.394 + 813.795 CMJheight (cm)	0.264	0.253	8447.1	<0.001
	−5274.272 + 7.944 CMJPpeak (W)	0.312	0.302	8162.4	<0.001
	−24,269.972 + 1054.434 Relative CMJPpeak (W·kg^−0.67^)	0.297	0.287	8251.9	<0.001
	−3981.616 + 358.575 1RM BP (kg)	0.379	0.370	7756.2	<0.001
	7822.102 + 15,511.486 Relative 1RM BP (kg/kg)	0.145	0.133	9099.0	<0.001
	1445.372+ 524.682 HS (kg)	0.268	0.257	8422.3	<0.001

**Notes:** R2: coefficient of determination value; SEE: standard error of the estimate; *p* = significance level; 1RM BP: 1 repetition maximum bench press; AS: armspan; BH: body height; BM: body mass; BMI: body mass index; CMJ: countermovement jump; HS: handgrip strength; Ppeak: peak power.

**Table 6:** Simple linear regression models to estimate main Hook punch impact power performance from anthropometric characteristics muscle power and strength performance in male boxing practitioners (N = 69).

	Equations	R2	Adjusted R2	SEE	*p*
Hook punch	−91,200.131 + 68,222.147 BH (cm)	0.340	0.330	8161.524	<0.001
	−75,519.506 + 57,602.673 AS (cm)	0.302	0.292	8390.310	<0.001
	5871.014 + 277.947 BM (kg)	0.131	0.118	9363.161	0.002
	27,934.788 + 73.270 BMI (kg·m^−2^)	0.005	−0.014	10,041.853	0.827
	4165.579 + 719.489 CMJheight (cm)	0.198	0.186	8996.800	<0.001
	−363.381 + 8.011 CMJPpeak (W)	0.305	0.295	8374.168	<0.001
	−12,883.602 + 914.257 Relative CMJPpeak (W·kg^−0.67^)	0.215	0.203	8902.529	<0.001
	−3729.337 + 424.385 1RM BP (kg)	0.510	0.503	7031.757	<0.001
	10,484.116 + 18,104.241 Relative 1RM BP (kg/kg)	0.190	0.178	9039.635	<0.001
	703.722 + 670.154 HS (kg)	0.420	0.411	7651.952	<0.001

**Notes:** R2: coefficient of determination value; SEE: standard error of the estimate; *p* = significance level; 1RM BP: 1 repetition maximum bench press; AS: armspan; BH: body height; BM: body mass; BMI: body mass index; CMJ: countermovement jump; HS: handgrip strength; Ppeak: peak power.

## Data Availability

Data generated or analyzed during this study are available from the corresponding author upon reasonable request.
